# A generalizable definition of chemical similarity for read-across

**DOI:** 10.1186/s13321-014-0039-1

**Published:** 2014-10-18

**Authors:** Matteo Floris, Alberto Manganaro, Orazio Nicolotti, Ricardo Medda, Giuseppe Felice Mangiatordi, Emilio Benfenati

**Affiliations:** 1 CRS4 Biomedicine, Parco Polaris, Loc. Pixinamanna, Pula, Italy; 2 Istituto di Ricerca Genetica e Biomedica (IRGB), CNR, Monserrato, Italy; 3 IRCCS-Istituto di Ricerche Farmacologiche Mario Negri, Milan, Italy; 4 Dipartimento di Farmacia - Scienze del Farmaco, Università degli Studi di Bari “Aldo Moro”, Bari, Italy

**Keywords:** Chemical similarity, Read-across, Applicability domain, QSAR

## Abstract

**Background:**

Methods that provide a measure of chemical similarity are strongly relevant in several fields of chemoinformatics as they allow to predict the molecular behavior and fate of structurally close compounds. One common application of chemical similarity measurements, based on the principle that similar molecules have similar properties, is the read-across approach, where an estimation of a specific endpoint for a chemical is provided using experimental data available from highly similar compounds.

**Results:**

This paper reports the comparison of multiple combinations of binary fingerprints and similarity metrics for computing the chemical similarity in the context of two different applications of the read-across technique.

**Conclusions:**

Our analysis demonstrates that the classical similarity measurements can be improved with a generalizable model of similarity. The proposed approach has already been used to build similarity indices in two open-source software tools (CAESAR and VEGA) that make several QSAR models available. In these tools, the similarity index plays a key role for the assessment of the applicability domain.

## Background

Methods that provide a measure of similarity between chemical compounds are becoming increasingly important, as several fields of chemoinformatics are in need of automated tools for the quick retrieval of congeneric molecules, thereby avoiding the use of human experts for the highly time demanding burden of checking every single compound and of evaluating its similarity with respect to a given reference [1]. Such a task is more challenging or even unfeasible when dealing with large-sized database comprising thousands of compounds.

To date, several approaches and algorithms for calculating chemical similarity have been developed [2],[3]. However, a still open and debated issue behind such different approaches is precisely in the *concept of similarity*. It is not possible to define in an unambiguous way (and, consequently, with an unambiguous algorithm) how similar two chemical entities are. In fact, two compounds can be seen as more or less similar with respect to the chemical features taken into consideration or chosen as a priority. For instance, if a similarity measurement is needed for QSAR purposes, the same molecular descriptors (i.e. physicochemical substituent representing hydrophobic, electronic and steric effects) used for deriving the QSAR model could be used. However, in other circumstances, the similarity could rely on holistic approaches based on a broader description of the chemical structure.

Another point leading to different approaches is related to practical applications of the similarity measurement. Excessive complexity must be avoided to obtain algorithms that can be calculated in a reasonable time.

The binary fingerprint approach is probably one of the most used methods to evaluate similarity [4],[5]. It is a milestone example of an acceptable trade-off between the wealth of information encoded and the chance of performing an easy and quick comparison of a large molecular data set.

A fingerprint consists of a fixed length string of bits in which the occurrence of molecular fragments is encoded (as one or more bits set to 1) by a hashing algorithm. The encoded sets of bits for different fragments could share one or more bits, so each bit of the fingerprint does not represent a unique structural feature (also meaning that it is not possible to generate the set of original fragments from a fingerprint). Fingerprints of two molecules can be compared to quantify (dis)similarity using some distance measure. A popular example is the Tanimoto index [6]. Structural keys represent a related approach: the string is not built with a hashing algorithm, but each bit represents an a-priori defined structural feature [7].

Fingerprints and structural keys are really useful for fast matching of similar structures and have been largely used for screening large molecular databases. Nevertheless, they suffer of some drawbacks [8],[9]. For instance, they encode the presence or absence of certain fragments or functional groups without accounting of their actual occurrence *per* compound (i.e. the number of times each fragment or function groups is found in the same molecule). This can lead to inaccurate matching, and thus can return artifacts.

Several binary fingerprints are available. Among others, fragment-based Daylight [10] and Tripos UNITY 2D fingerprints [11] are some of the best known commercial examples.

In the present work, we decided to focus our attention only on the fingerprints available in the Chemistry Development Kit [12],[13], which are free and open source implementations of different fingerprint algorithms.

Furthermore, several similarity coefficients are available; a comprehensive and up-to-date list has been recently summarized by Todeschini et al. [14] and used in the present work to choose similarity coefficients to be tested. Remarkably, Todeschini listed 51 similarity coefficients for binary variables extracted from the literature and compared using both simulated and real data.

Our aim is that of exploring the possibility of blending fingerprints with non-binary structural keys based on constitutional molecular descriptors. The basic idea is that such a combination can help to overcome the drawbacks of a plain fingerprint approach and thus to increase the accuracy of similarity measurements, yet avoiding an excessive calculation complexity. In this respect, we developed an integrated similarity index resulting from the weighted combination of a fingerprint array and three structural keys based on molecular descriptors. We then designed a batch process to evaluate the performances of different fingerprints, different similarity coefficients, and different weighting schemes for the elements contained in the final index.

We chose to use, for the batch process, a *read-across approach* on two distinct datasets, in order to find an acceptable criterion of choice of elements and weighting scheme for the similarity index in a generic application. Our efforts resulted in a comparative analysis of the performances on the two datasets of all the possible combinations of 9 fingerprint implementations and 44 similarity coefficients, followed by an exploration of a reasonable subset of all the possible weighting schemes for the fingerprint and the structural keys based on molecular descriptors.

A scheme providing good performances on both datasets has finally been chosen to build the similarity index, actually implemented in the VEGA platform [15] (an open-source on-line platform providing several QSAR models).

## Methods

### Fingerprints

We decided to evaluate the performance of 9 different fingerprint algorithms, which are implemented in the Chemistry Development Kit (CDK) libraries. While they fall under the generic definition of fingerprints, some of them are structural keys and not hashing-based fingerprints. More specifically, the fingerprints here considered are the following:

1. Default Fingerprints (as defined by Daylight [10]),
2. Extended Fingerprints (same as Default, but with additional bits that take into account ring features),
3. Graph-only fingerprints (same as Default, but do not take bond orders into account),
4. Hybridization fingerprints (same as Default, but do not perform aromaticity perception),
5. E-State fragments (79 bit fingerprints described by Kier and Hall [16]),
6. Klekota-Roth fingerprints (set of 4860 chemical substructures enriched for biological activity [17]),
7. MACCS keys (structural key made of a set of 166 bits [18]),
8. Pubchem fingerprints (structural key made of 881 keys [19]),
9. Substructure fingerprints (structural key made of 307 bits [20].

### Molecular descriptors based structural keys

We decided to build three structural keys made of molecular descriptors related to constitutional issues. The hypothesis that lead to these keys was to test if such information could be successfully coupled with the use of fingerprints, so that these keys can fill the information gap of fingerprints. As these keys are made of molecular descriptors, they are no longer binary keys. The descriptors used for these keys were calculated by an in-house JAVA software module, based on CDK libraries; for the definition of the descriptors the commercial software Dragon [21] has been taken as reference.

The three keys are:

Constitutional descriptors (CD): this key is made of 35 constitutional descriptors, as reported in Table 1

**Table 1:** **Descriptors in the constitutional descriptors (CD) key**

Name	Description
MW	Molecular weight
AMW	Average molecular weight
Sv	Sum of atomic van der Waals volumes
Mv	Mean atomic van der Waals volum
Sp	Sum of atomic polarizabilities
Mp	Mean atomic polarizability
Se	Sum of atomic Sanderson electronegativities
Me	Mean atomic Sanderson electronegativity
nAt	Number of atoms
nSk	Number of non-H atoms
nBt	Number of bonds
nBo	Number of non-H bonds
nBm	Number of multiple bonds
nDblBo	Number of double bonds
nTrpBo	Number of triple bonds
nArBo	Number of aromatic bonds
SCBO	Sum of conventional bond orders (H-depleted)
nH	Number of Hydrogen atoms
nC	Number of Carbon atoms
nN	Number of Nitrogen atoms
nO	Number of Oxygen atoms
nP	Number of Phosphorous atoms
nS	Number of Sulfur atoms
nF	Number of Fluorine atoms
nCl	Number of Chlorine atoms
nBr	Number of Bromine atoms
nI	Number of Iodine atoms
nB	Number of Boron atoms
HPerc	Percentage of H atoms
CPerc	Percentage of C atoms
NPerc	Percentage of N atoms
OPerc	Percentage of O atoms
XPerc	Percentage of halogen atoms
nHet	Number of heteroatoms
nX	Number of halogen atoms

Hetero-atoms descriptors (HD): this key is made of 11 counters for different types of hetero-atoms, as reported in Table 2. These descriptors are a subset of the constitutional descriptors. We chose to build a key with this subset in order to have the possibility of giving it different weights so to remark the feature it represent in the computation of chemical similarity. This stems from the observation that often the generic idea of chemical similarity is strongly influenced by small differences in the number and type of heteroatoms, i.e. molecules with several similar features (molecular weight, number and type of rings, bonds etc.) can be considered remarkably different just because they differ in the presence/absence of some heteroatoms.

**Table 2:** **Descriptors in the hetero-atoms descriptors (HD) key**

Name	Description
nN	Number of Nitrogen atoms
nO	Number of Oxygen atoms
nP	Number of Phosphorous atoms
nS	Number of Sulfur atoms
nF	Number of Fluorine atoms
nCl	Number of Chlorine atoms
nBr	Number of Bromine atoms
nI	Number of Iodine atoms
nB	Number of Boron atoms
nHet	Number of heteroatoms
nX	Number of halogen atoms

Functional Groups (FG): this key is made of 154 functional groups, as defined in Dragon.

### Similarity coefficients

We built two sets of similarity coefficients to be tested respectively with the chosen fingerprints (binary coefficients) and descriptors based keys (non-binary coefficients). The chosen binary coefficients are 44, reported in Table 3, coming from the work of Todeschini et al. [14]. The chosen non-binary coefficients are 6, reported in Table 4, coming from the work of Holliday [22]. All the coefficients have been implemented in an in-house JAVA software module.

**Table 3:** **Binary similarity coefficients**

No.	Name	No.	Name
1	Simple matching	23	Dennis
2	Rogers/Tanimoto	24	Cole 1
3	Jaccard/Tanimoto	25	Cole 2
4	Gleason/Dice/Sorensen/Nei-Li	26	Dispersion
5	Russel-Rao	27	Goodman-Kruskal
6	Forbes	28	Sokal-Sneath 3
7	Simpson	29	Sokal-Sneath 4
8	Braun-Blanquet	30	Phi
9	Driver-Kroeber/Ochiai	31	Dice 1
10	Baroni-Urbani 1	32	Dice 2
11	Kulczynski 1	33	Sorgenfrei
12	Sokal-Sneath 1	34	Cohen
13	Sokal-Sneath 2	35	Peirce 1
14	Jaccard 2	36	Peirce 2
15	Faith	37	Maxwell-Pilliner
16	Mountford	38	Harris-Lahey
17	Michael	39	CT1
18	Rogot-Goldberg	40	CT2
19	Hawkins-Dotson	41	CT3
20	Yule 1	42	CT4
21	Yule 2	43	CT5
22	Fossum	44	Austin-Colwell angular coeff.

The number of each coefficient is the same as in the paper by Todeschini et al.

**Table 4:** **Non-binary similarity coefficients**

No.	Name	Code
1	Mean Camberra	MC
2	Divergence	Div
3	Bray/Curtis	BC
4	Dice	Dice
5	Sokal/Sneath	SS1
6	Cosine/Ochiai	Cos

The code of each coefficient is the same as in the paper by Holliday et al.

### Similarity index

In order to combine the fingerprint with the descriptors based keys, we designed a generic scheme for the similarity index SI, defined as follow:

1 $$\mathrm{SI}\left(A,B\right)\;=\;\left[\mathrm{Sb}\left({\mathrm{FP}}_{a},{\mathrm{FP}}_{b}\right)\right]W_{\mathrm{fp}}\;*\;\left[\mathrm{Snb}\left({\mathrm{CD}}_{a},{\mathrm{CD}}_{b}\right)\right]W_{\mathrm{cd}}\;*\;\left[\mathrm{Snb}\left({\mathrm{HD}}_{a},{\mathrm{HD}}_{b}\right)\right]W_{\mathrm{hd}}\;*\;\left[\mathrm{Snb}\left({\mathrm{FG}}_{a},{\mathrm{FG}}_{b}\right)\right]W_{\mathrm{fg}}$$

where:

A and B are two molecules to be compared;

FP_a_, CD_a_, HD_a_, FG_a_, FP_b_, CD_b_, HD_b_, FG_b_ are the Fingerprint, Constitutional Descriptors, Heteroatom Descriptors and Functional Groups keys as defined before, respectively calculated on the two molecules A and B;

Sb(X_a_,X_b_) is the result of the application of a binary similarity coefficient to two fingerprints X_a_ and X_b_, where the resulting values are in the interval [0,1];

Snb(X_a_,X_b_) is the result of the application of a non-binary similarity coefficient to two descriptors based keys X_a_ and X_b_, where the resulting values are in the interval [0,1];

W_fp_, W_cd_, W_hd_, W_fg_ are the relative weights of the four contributions, under the condition:

2 $$W_{\mathrm{fp}}\;+\;W_{\mathrm{cd}}\;+\;W_{\mathrm{hd}}\;+\;W_{\mathrm{fg}}\;=\;1$$

As it can been seen, the proposed index simply takes into account the different contribution of the similarity (calculated with the chosen coefficient), each one with a given weight.

### Datasets and read-across model

We chose two publicly available datasets from the VEGA project. The bioconcentration factor in fish (BCF) dataset comprises 473 compounds with the experimental BCF values. The water/octanol partition coefficient (LogP) dataset consists of 10,005 compounds with the experimental logP values.

The choice of testing the Similarity Index on these two datasets arises from the goal of finding a setting for the SI that potentially could give good performances on different kinds of data, thus implementing a “generic” idea of chemical similarity. In more detail, we focused our analysis on an endpoint with relevance for toxicity (BCF) and on a physical-chemical property (logP) with several applications, furthermore having markedly different size (BCF: 860 molecules; logP 10005 molecules).

For the purpose of testing the performances of the proposed Similarity Index with different settings, we implemented in an in-house JAVA module a simple *read-across based prediction model*, where a property is predicted for a given compound by finding the three most similar compounds of the dataset according to the SI, and calculating the mean of their three experimental values, weighted by their SI values.

In our procedure, we calculated predictions on the basis of the *leave-one-out* strategy adopted for cross-validation. Iteratively, one molecule at a time was left out of the dataset to be predicted using our read-across approach on the remaining molecules.

Finally, as the above described model approach is analogous to a regression model, we calculated the values of the coefficient of determination (R^2^) and of the root mean square error (RMSE) on all the predictions of the dataset, and used these values to quantify the quality of the model, that is directly related to how good the SI settings are.

### Evaluation process

We applied a combinatorial strategy to test all the possible permutation of different settings (similarity coefficient, binary fingerprints, non-binary descriptors, weighting scheme), calculating for each of these settings the read-across model for the two datasets and the resulting R^2^ and RMSE.

In a preliminary step, we processed both datasets with all the combinations of the different fingerprints and of binary similarity coefficients, for a total of about 400 permutations. At this level, we selected the best combinations (based on R^2^ and RMSE).

We then performed a second analysis where we used the selected couple of fingerprint/coefficient and a set of combinations of the weights for the SI contributions and of non-binary similarity coefficients for the descriptors keys. We chose the following ranges for the weights:

W_fp_: between 0.3 and 1.0, with steps of 0.1

W_cd_: between 0.0 and 0.4, with steps of 0.05

W_hd_: between 0.0 and 1.0, with steps of 0.05

W_fg_: between 0.0 and 1.0, with steps of 0.05

under the usual condition of having the sum of weights equal to one. The batch process generated a total of about 7200 combinations of weights/coefficient.

## Results and discussion

The first step has been to analyze the results of all possible permutations of fingerprint types and similarity coefficients, in order to find the best combination to be used in the following step.

We evaluated simultaneously the results by considering the values of both R^2^ and RMSE, using two objectives known as *utility function* and *desirability function* for ranking the combinations on the basis of the performances on both the datasets. Such functions are usually applied in the field of multi-criteria decision making [23],[24]. The two functions have been calculated as:

3 $$\mathrm{DES}\;=\;\left(R_{\mathrm{bcf}}\right)0.25*\left({\mathrm{RMSE}}_{\mathrm{bcf}}\right)0.25*\left(R_{\mathrm{logp}}\right)0.25*\left({\mathrm{RMSE}}_{\mathrm{logp}}\right)0.25$$

4 $$\mathrm{UTI}\;=\;0.25*R_{\mathrm{bcf}}+0.25*{\mathrm{RMSE}}_{\mathrm{bcf}}+0.25*R_{\mathrm{logp}}+0.25*{\mathrm{RMSE}}_{\mathrm{logp}}$$

where R_bcf_ and R_logp_ are the R^2^ values obtained respectively on the BCF and on the LogP datasets, and RMSEbcf and RMSElogp are the RMSE values obtained respectively on the BCF and on the LogP datasets. Both functions are calculated after scaling the four parameters in the range [0,1] and transforming RMSEbcf and RMSElogp in their respective complements. Thus, all the four parameters had values in the range [0,1] where values towards 1 mean optimality. Importantly, both DES and UTI returned values in the range [0,1], such values have been used to rank all the permutations, with higher values flagging better solutions.

Noteworthy, the rankings obtained from the desirability and the utility functions had exactly the same sorting for the top ten solutions, as reported in Table 5.

**Table 5:** **Best ten results for fingerprints/similarity metrics combinations**

FP	Metrics	BCF R^2^	BCF RMSE	LogP R^2^	LogP RMSE	DES	UTI
Extended	37	0.546	0.917	0.775	0.872	0.970	0.971
Extended	34	0.546	0.919	0.776	0.870	0.970	0.970
Extended	18	0.542	0.922	0.777	0.869	0.965	0.965
Pubchem	28	0.541	0.906	0.772	0.870	0.963	0.963
Extended	42	0.534	0.919	0.780	0.858	0.961	0.962
Default	18	0.549	0.913	0.766	0.890	0.954	0.955
Extended	13	0.541	0.913	0.770	0.875	0.954	0.954
Default	34	0.549	0.913	0.765	0.891	0.953	0.953
Extended	1	0.540	0.917	0.770	0.876	0.950	0.950
Default	37	0.549	0.913	0.764	0.893	0.950	0.950

FP stands for the fingerprint type, Metrics for the number (id) of the binary similarity coefficient (as reported in Table 3), for the R2 correlation coefficient, RMSE for the root mean square error, DES for the desirability function, UTI for the utility function.

The fingerprints found in the ten best solutions are the Extended Fingerprints, Pubchem Key and Default Fingerprint. It is interesting to note that two different approaches emerged as best solutions, as the Default and Extended fingerprints are strictly related, while Pubchem is a structural key.

For the fingerprints, it is not surprising that the Extended yield better results than the Default, as Extended are the same as default with the extension of extra bits encoding information about rings. Other fingerprints, that are similar to the Default but contain less (more generic) information such as Graph-Only or Hybridization disclose far more worse results. The best coefficients found in combination with the fingerprints are 37 (Maxwell-Pilliner), 34 (Cohen), 18 (Rogot-Goldberg), 42 (CT4), 13 (Sokal-Sneath), 1 (simple matching).

The Pubchem key appears in the best solutions only once, combined with the similarity coefficient no. 28 (Sokal-Sneath 3).

In the second step, having selected the Extended fingerprints and the coefficient no. 37 (Maxwell-Pilliner) as the best solutions, a number of about 7200 combinations of weights and non-binary similarity coefficients has been analyzed. Similarly to the the first step, we calculated the utility and desirability values considering both the R^2^ and RMSE of the read-across approach on the two datasets, and ranked the combinations. Both the rankings obtained from the desirability and the utility functions are equal with respect to the top ten best solutions, reported in Table 6.

**Table 6:** **Best ten results for keys weights/similarity metrics combinations**

W_fp_	W_hd_	W_cd_	W_fg_	Metrics	BCF R^2^	BCF RMSE	LogP R^2^	LogP RMSE	DES	UTI
0.4	0.1	0.35	0.15	3	0.63	0.83	0.87	0.68	0.996	0.996
0.3	0.15	0.35	0.2	3	0.62	0.84	0.87	0.67	0.996	0.996
0.3	0.15	0.3	0.25	3	0.62	0.84	0.87	0.68	0.993	0.993
0.3	0.1	0.35	0.25	3	0.62	0.84	0.87	0.67	0.992	0.992
0.3	0.2	0.35	0.15	3	0.62	0.84	0.87	0.67	0.992	0.992
0.3	0.2	0.3	0.2	3	0.62	0.84	0.87	0.68	0.991	0.991
0.3	0.2	0.25	0.25	3	0.62	0.84	0.87	0.69	0.989	0.989
0.4	0.15	0.3	0.15	3	0.62	0.83	0.86	0.70	0.989	0.989
0.4	0.1	0.3	0.2	3	0.62	0.84	0.86	0.69	0.988	0.988
0.3	0.05	0.35	0.3	3	0.61	0.85	0.87	0.67	0.988	0.988

W_xx_ stands for the weights of the different keys contributions (FP, HD, CD, FG, as defined in the article), Metrics for the number (id) of the non-binary similarity coefficient (as reported in Table 4), for the R2 correlation coefficient, RMSE for the root mean square error, DES for the desirability function, UTI for the utility function.

A first result is that all the ten best solutions use the coefficient no. 3 (Bray-Curtis) for the measurement of the non-binary keys of descriptors. Subsequently, it can be easily observed that all the ten solutions have a similar distribution of the weight values. In the best solution the fingerprints block represents the most important contribution (weight of 0.4), followed by the Constitutional Descriptors block (0.35), the Functional Groups Descriptors block (0.15) and the Heteroatoms Descriptors block (0.1). This result can be interpreted as follows:

The SI is mainly constituted by the classical fingerprint-based comparison, strongly corrected with some constitutional information like number (and type) of atoms and number (and type) of bonds; this part of the SI could be considered as the core contribution to generalizability of the SI.

A smaller contribution of functional and heteroatoms descriptors is required to extend the information embedded in the fingerprint and constitutional descriptor blocks; we would consider this block as the part of SI which explains the “fine chemical differences” within the dataset.

## Conclusions

The computation of similarities between chemical compounds is usually based on the use of common binary representations of chemical structures (i.e. 2D fingerprints) and a similarity coefficient (usually the Tanimoto distance). It has been recently demonstrated by Todeschini et al. [14] that other similarity coefficients perform better than the Tanimoto distance in terms of effectiveness for similarity-based virtual screening using simulated and real datasets. With our work, we demonstrated how achieve a higher accuracy in measures of chemical similarity by combining fingerprints with non-binary structural keys based on constitutional molecular descriptors. The basic idea is that such a combination can resolve the drawbacks of a plain fingerprint approach. Thus, we built a combined similarity index, where a fingerprint and 3 molecular descriptors based structural keys are combined with different weights. We then designed a combinatorial process to evaluate the performances of different fingerprints, different similarity coefficients, and different weighting schemes for the elements of the final index, in the context of two heterogeneous datasets.

## Authors’ contributions

Conceived and designed the experiments: EB, MF and AM. Performed the experiments: MF. Wrote the paper: MF, AM and ON. All authors analyzed the data, discussed the results and commented on the manuscript. All authors have given approval to the final version of the manuscript.
